# Determination of *Salmonella* spp., *E. coli* VTEC, *Vibrio* spp., and Norovirus GI-GII in Bivalve Molluscs Collected from Growing Natural Beds in Sardinia (Italy)

**DOI:** 10.3390/foods6100088

**Published:** 2017-10-11

**Authors:** Marta Marceddu, Sonia Lamon, Simonetta G. Consolati, Sara Ciulli, Roberta Mazza, Anna Mureddu, Domenico Meloni

**Affiliations:** 1 Dipartimento di Medicina Veterinaria, Università degli Studi di Sassari, Via Vienna 2, 07100 Sassari, Italy; mmarceddu@uniss.it (M.M.); sonialamon@gmail.com (S.L.); sgconsolati@uniss.it (S.G.C.); rmazza@uniss.it (R.M.); anna.mureddu@tin.it (A.M.); 2 Dipartimento di Scienze Mediche Veterinarie, Alma Mater Studiorum, Università di Bologna, Via Tolara di Sopra 50, 40064 Ozzano dell'Emilia, Italy; sara.ciulli@unibo.it

**Keywords:** shellfish, *E. coli* VTEC, *Vibrio* spp., Norovirus

## Abstract

The aim of the present study was to evaluate the presence of *Salmonella* spp., verotoxigenic *E. coli* (VTEC), *Vibrio* spp., and Norovirus GI-GII in bivalve molluscs, cockles, and European grooved carpet shells (*Cerastoderma* spp. and *Ruditapes decussatus*) collected from a class B growing natural bed in Sardinia (Italy). All of the samples were analysed for *Salmonella* spp. detection according to European Commission Regulation (EC) 2285/2015. Detection and enumeration of *Vibrio* spp. were performed according to previously published methods. Presumptive identification of *Vibrio* spp. isolates was performed by means of conventional biochemical tests. *E. coli* VTEC was isolated following a direct multiplex polymerase chain reaction (PCR) screening test. Norovirus GI and GII were determined by reverse transcriptase-polymerase chain reaction (RT-PCR). No *Salmonella* spp. were detected. The prevalence of *Vibrio* spp. was 90%, and the average contamination levels were 3.19 ± 1.07 and 2.84 ± 0.31 Log_10_ cfu/g in cockles and European grooved carpet shells, respectively. The prevalence of *E. coli* VTEC was 6.6%. All of the isolates showed a complete pathogenicity profile. The presence of Norovirus was highlighted in 25% of European grooved carpet shells samples. Results showed the typical microbiological profile of bivalve molluscs collected from backwaters and confirmed the capability of shellfish to accumulate *E. coli* VTEC, pathogenic vibrios, and Norovirus. The presence of such pathogens in shellfish is of major concern for the safety of consumers.

## 1. Introduction

Foodborne zoonoses associated with the consumption of bivalve molluscs are reported worldwide [1,2,3,4]. Shellfish contamination occurs because they are suspension feeders that selectively filter and concentrate small particles of phytoplankton, zooplankton, and the contaminant substances associated with them: bacteria of faecal origin and pathogens adapted to the marine environment, viruses, algal biotoxins, and chemicals [5,6,7,8]. Viruses and naturally occurring vibrios are the most often cited causative agents of disease and death related to shellfish consumption [1,5,9]. Although *Salmonella* spp. is considered one of the most common causes of human gastroenteritis [10], the risk of foodborne diseases associated with mollusc consumption is low compared to viruses and naturally occurring vibrios [11]. The incidence of foodborne outbreaks associated with the consumption of shellfish contaminated with Norovirus and pathogenic vibrios is increasing, although in many European countries production control plans are being implemented [7,12,13]. According to current European Legislation (Council Regulation (EC) 2285/2015), the evaluation of shellfish safety is based entirely on the use of *E. coli* as an indicator of faecal contamination. Historically, faecal indicator bacteria including total and faecal coliforms and enterococci have been used in many countries as a monitoring tool and for the prediction of the presence of bacterial, viral, and protozoan pathogens. These microorganisms are of faecal origin from higher mammals and birds, and their presence in water may indicate faecal pollution and possible association with enteric pathogens [8]. Several studies [14,15,16] have shown that faecal indicators provide an inadequate index of microbiological safety and are poorly predictive of the presence of microorganisms adapted to the aquatic environment (*Vibrio* spp.) and other pathogens detected in limited numbers (Noroviruses, *E. coli* O157). Norovirus (NoV) causes self-limiting infections characterised by gastrointestinal symptoms that have an average incubation time of 36 h; it generally lasts for about 48 h and resolves spontaneously without complications. Although viral gastroenteritis has low mortality rates (0.1%), the annual incidence recorded in some countries makes relevant economic and social costs and public health in general. However, the absence of epidemiologic episodes suggests that the number of reported cases represents an underestimation of the true incidence, especially in terms of medium or small forms that do not require recourse to hospitalisation [17]. Among pathogenic microorganisms adapted to the marine environment and involved in episodes of human infection, naturally occurring pathogenic vibrios, as *V. parahaemolyticus*, *V. cholerae*, and *V. vulnificus* are the most important [18]. The prevalence of such microorganisms in molluscs is variable and is strongly related to the heterogeneity of the microbiological methods, which differ mainly due to incubation temperatures (20–25 °C or 37 °C). Several authors reported a prevalence of *Vibrio* spp. in seafood samples collected in Italy, ranging from 34% to 93% [9,19,20]. Verotoxigenic *E. coli* (VTEC) is not commonly found in fishery products, but it has been documented that shellfish harvested in areas contaminated by landfill can carry VTEC and enterotoxigenic *E. coli* (ETEC) [8]. Generally, they are isolated from the gastrointestinal tract of ruminants, which are recognised as the main reservoirs [21]. A significant number of human infections due to *E. coli* are caused by serotype O157, mainly through the consumption of contaminated food, but also by contact with the environment and/or the use of water [22]. VTECs, predominantly O157, have been isolated in breeding environments from different sites such as soil, manure, sewage, drinking water, irrigation water, crops, and various equipment [8]. From agricultural environments, they can be transferred to watercourses, especially during periods of high rainfall, and subsequently spread to coastal areas [23]. Shellfish harvested in these areas can consequently concentrate the pathogen and thereby pose a risk to the health of consumers. In Italy, bivalve molluscs represent the most important aquaculture resource, representing over the half of total national production, mainly composed by Manila clams and Mediterranean mussels [22]. Nowadays, Italy is the leading European producer of Manila clams and the second in the world after China [24,25,26]. In Sardinia, the regional shellfish sector is well consolidated: the annual production accounts for 83% of the aquaculture species and rests almost exclusively on Mediterranean mussel [27]. Alongside aquaculture areas, cockles (*Cerastoderma* spp.) and European grooved carpet shells (*Ruditapes decussatus*) are native species typical of growing natural beds in several lagoons of remarkable naturalistic value, where artisanal fishing is currently practiced. These bivalve molluscs are harvested mainly for local consumption and are often consumed raw or lightly cooked. Recent literature on the occurrence of *E. coli* VTEC, *Salmonella* spp., *Vibrio* spp., and Norovirus in the Sardinian bivalve molluscs supply chain is limited. Therefore, the aim of the present study was to evaluate their presence in *Cerastoderma* spp. and *Ruditapes decussatus* collected from a class B growing natural bed.

## 2. Materials and Methods

### 2.1. Sampling

The survey was conducted on samples (*n* = 40) from four batches of cockles (*n* = 20) and grooved carpet shells (*n* = 20) from class B growing natural bed present in the lagoons of the Oristano province (Sardinia, Italy). A batch is a quantity of live bivalve molluscs collected from the same production area and subsequently intended for delivery to an approved dispatch centre, purification centre, relaying area, or processing plant as appropriate. Five samples per batch were collected after the purification treatment. The purification centre was “recirculating”: bivalve molluscs were placed in high density polyethylene (HDPE) tanks stacked on top of each other and supplied by a common seawater source in parallel. The flow of disinfected water by ultraviolet light (UV) was introduced into the tank by means of a spray bar onto the surface of the water. Before the disinfection of water, additional treatments including protein skimmers and biofilters were applied to recirculated seawater to reduce the concentrations of proteins and ammonia from shellfish.

### 2.2. Microbiological Analysis

All the samples were shipped, refrigerated, to the laboratories of the Dipartimento di Medicina Veterinaria, Università degli Studi (Sassari) in insulated boxes, and were submitted to microbiological analysis within 24 h of harvesting. Samples were processed for microbiological detection, enumeration, and graduated dilutions according to the ISO 6887-1: 2004 method.

#### 2.2.1. Detection of *Salmonella* spp.

All the samples were analysed for *Salmonella* spp. detection according to the Official International Organisation for Standardisation (ISO) cultural methods, UNI EN 6579:2002. Briefly, 25 g of each sample were added to 225 mL of Buffered Peptone Water (BPW) and incubated at 37 °C for 18 h (Thermo Fisher Scientific, Waltham, MA, USA). Following incubation, 100 μL of the BPW enrichment were inoculated in 10 mL of Rappaport-Vassiliadis soya enrichment broth (RVS) and incubated at 42 °C for 24 h while 100 μL of the BPW enrichment were streaked over the surface of a Modified Semi-Solid Rappaport-Vassiliadis agar plate (MSRV) and incubated at 42 °C for 24 h. Finally, 1 mL of the BPW enrichment was transferred to 10 mL of Muller-Kaufmann tetrathionate-novobiocin broth (MKTTn) amended with iodine and novobiocin. Following incubation, RVS, MKTTn, and MSRV were subcultured onto the surface of one Xylose-Lysine-Desoxycholate (XLD) agar plate to obtain well-isolated colonies, incubated at 37 °C for 24 h. After incubation, XLD plates were examined for characteristic typical salmonella-like colonies with black centres and a light transparent zone of reddish colour.

#### 2.2.2. Detection of *E. coli* VTEC

For the direct determination of *E. coli* VTEC, 10 g of each sample were subjected to selective enrichment in 90 mL of modified-Tryptone Soya Broth (m-TSB) containing novobiocin (20 mg/L) and incubated at 37 °C for 18–20 h. Subsequently, an aliquot was frozen at −20 °C (Angelantoni Industrie Spa, Massa Martana, Italy) for immuno-magnetic separation (IMS) screening test by using the protocol for the *E. coli* O157, O26, O103, O111, and O145 Dynabeads capture (Dynal, Oslo, Norway), as described by the manufacturer. Another aliquot equal to 1 mL was used for DNA extraction using the Chelex 100 (BioRad, Hercules, CA, USA) resins. VTEC detection was carried out by a polymerase chain reaction (PCR) one-step method for the determination of *stx*1 and *stx*2 genes using the primer sets MK1/MK2 [28]. A negative control (NCTC 12900) and a positive control (ATCC 35150) were included at each PCR test. All PCR amplifications were performed by using a GeneAmp PCR System 9700 (Applied Biosystems, Foster City, CA, USA). All PCR-positive samples (VTEC presence) were subjected to qualitative determination of *E. coli* O157, O26, O103, O145, and O111 serogroups by IMS using Dynabeads anti-*E. coli* (Invitrogen, Carlsbad, CA, USA). Each Dynabeads-microorganism complex was streaked on CT-SMAC (MacConkey agar cefixime tellurite sorbitol, Thermo Fisher Scientific Oxoid Ltd., Basingstoke, UK) for the detection of serogroup O157, CT-RMAC (MacConkey agar cefixime tellurite rhamnose, Thermo Fisher Scientific Oxoid Ltd, Basingstoke, UK) for O26, and EHLY (Enterohemolysin agar, Thermo Fisher Scientific Oxoid Ltd, Basingstoke, UK) for serogroups O103, O111, and O145. All of the plates were incubated at 37 °C for 24 h. Isolates with typical morphological characteristics were subjected to biochemical identification by the API^®^ 20E identification system (bioMérieux, Craponne, France). All of the isolated *E. coli* were subjected to a multiplex PCR for the detection of *stx*1, *stx*2, *hly*A, and *eae* genes [29].

#### 2.2.3. Detection and Enumeration of *Vibrio* spp.

Detection and enumeration of *Vibrio* spp. were carried out by plating 0.1 g of shellfish homogenate and its 10-fold dilutions over the surface of 3% NaCl thiosulfate–citrate–bile salts–sucrose agar plates (TCBS, Thermo Fisher Scientific Oxoid Ltd, Basingstoke, UK). The plates were incubated at 20 °C for 3–5 days [30]. Presumptive identification of *Vibrio* spp. isolates was performed by means of conventional biochemical tests: five colonies from the plates of each medium were randomly selected and streaked on 3% NaCl tryptone soya agar plates (TSA-s, Thermo Fisher Scientific Oxoid Ltd., Basingstoke, UK), and then incubated at 30 °C for 24 h. Colonies on TSA-s were successively screened by oxidase and catalase tests, Gram staining, sugar fermentation, and sensitivity to vibriostatic agent O129. Presumptive biochemical identification was carried out by the API identification system (bioMérieux, Craponne, France).

### 2.3. Virological Analysis

From pooled cockles and European grooved carpet shells samples, stomach and digestive diverticula were isolated by dissection to obtain 5–10 g of tissue. Viral RNA was extracted according to previously described methods [31]. NoV detection was carried out by a RT-PCR one-step method using the previously described primer sets JV13I and JV12Y [32]. Subsequently, NoV GI and GII were determined by two specific semi-nested RT-PCR [33,34].

## 3. Results

### 3.1. Microbiological Analysis

#### 3.1.1. Detection of *Salmonella* spp.

*Salmonella* spp. was not detected in 25 g of samples of cockles and European grooved carpet shells.

#### 3.1.2. Detection of *E. coli* VTEC

The direct PCR method used for the preliminary screening of VTECs showed a total prevalence of 6.6%. One sample of cockles and one of European grooved carpet shells exhibited a positive result during the same sampling session. However, conventional PCR is generally less sensitive than real-time PCR, so this could be an underestimation of frequency. Altogether, 10 *E. coli* strains were isolated: *n. 2* O157; *n. 2* O26; *n. 2* O103, *n. 2* O145, and *n. 2* O11. All of the isolates showed a complete pathogenicity profile (*stx*1 +, *stx*2 +, *eae* +, *hly*A +). The prevalence of naturally occurring vibrios was 90%, and the average contamination levels (mean ± standard deviation (s.d.)) were 3.19 ± 1.07 and 2.84 ± 0.31 Log_10_ cfu/g in cockles and European grooved carpet shells, respectively (Table 1).

#### 3.1.3. Detection and Enumeration of *Vibrio* spp.

The use of TCBS agar NaCl 3% with incubation at 20 °C allowed for the recovery of an abundance of *Vibrio* spp. in all of the samples. However, when used for direct plating, TCBS may underestimate the contamination levels [30]. Altogether, 36 *Vibrio* spp. isolates were presumptively identified by biochemical tests using the API 20 NE identification system (bioMérieux, Craponne, France). The prevalent species were *V. vulnificus* (98%) and *V. alginolyticus* (2%).

### 3.2. Virological Analysis

The presence of NoV was highlighted in 25% of the samples (European grooved carpet shells). Regarding the prevalence of the two genogroups, the results (25% GII-positive and 10% GI-positive) confirmed the higher circulation of GII with respect to GI in shellfish [10,35,36]. On the contrary, only two samples presented simultaneous contamination with both genogroups.

## 4. Discussion

The results of the present study showed the typical microbiological profile of bivalve molluscs collected from backwaters and confirmed the capability of shellfish to accumulate *E. coli* VTEC, Norovirus, and pathogenic vibrios. *Salmonella* spp. was not detected, and this result was not surprising; recent studies have shown that its presence is strongly related to the bivalve species considered, the classification areas in which molluscs were collected, and the sampling occasion [37]. Shellfish harvested from coastal areas near agricultural environments are at high risk of pollution of agro-zootechnical origin and may be contaminated by *E. coli* VTEC transferred from contaminated animals to watercourses [21]. However, the low prevalence of VTEC in shellfish could be related to the low number of these pathogens in the coastal environment, to the presence of competitive bacterial flora, and to the reduced in vitro vitality of these microorganisms in respect to the marine environment [38]. The direct determination of *E. coli* by PCR allowed us to detect the presence/absence of VTEC directly in the food matrices. However, some difficulties in isolating strains from selective media highlights the need to further optimise the operational protocols. Some problems related to the factors that may interfere with the vitality and survival capacity of the strains should be resolved. The presence of NoV in shellfish (25%) was not generally related to the presence of faecal bacteria. Moreover, their presence in polluted waters is very scarce due to the competitive action exerted by these bacteria [39]. A previous study carried out in several Italian regions showed NoV contamination in 51.5% of samples. Interestingly, the prevalence of NoV in samples collected in Sardinia was 62.1% [35]. The recovery of NoV GI and GII posed an important health significance, in relation to the ability of such viruses to withstand the environment for longer periods in presence of favorable conditions. As previously reported [3,40,41], the purification treatment, utilised worldwide to purge bivalve molluscs from faecal contaminants, was unsatisfactory with respect to seawater autochthonous vibrios. These results might be related to *Vibrio* spp. release dynamics in depuration [13,42]: all microbial species related to the aquatic habitat accumulate in the intestines of mussels where they can multiply, thus making the depuration systems ineffective [43,44]. *V. vulnificus* was the prevalent *Vibrio* spp. species: the presence of such pathogens in Sardinian bivalve molluscs is of major concern for the safety of consumers. In 2000–2001, two deaths caused by fulminant sepsis attributed to *V. vulnificus* were reported in the same production area. In both cases, subjects with severe chronic liver diseases manipulated and/or consumed contaminated bivalve molluscs a few days before death [45].

## 5. Conclusions

This survey is a practical contribution to the acquisition of data on the safety of bivalve molluscs (*Ruditapes decussatus* and *Cerastoderma* spp.) collected from class B growing natural beds in Sardinia (Italy). The presence of *E. coli* VTEC, pathogenic vibrios, and NoV in shellfish collected from natural environments, where artisanal fishing is currently practiced and these molluscs are consumed raw or lightly cooked, pose a significant risk to the health of consumers. Currently, in Regulation (EC) 2073/2005 on the microbiological criteria applicable to foodstuffs, food safety criteria related to the presence of *E. coli* VTEC, pathogenic vibrios, and NoV in shellfish are not present.

## Figures and Tables

**Table 1:** Enumeration (mean ± s.d.) of *Vibrio* spp. (Log_10_ cfu/g) in *Cerastoderma* spp. and *Ruditapes decussatus* after purification.

*Vibrio* spp.	Batch *				
	1	2	3	4	Mean
*Cerastoderma* spp.	3.76 ± 1.45	2.74 ± 0.37	2.86 ± 0.25	3.40 ± 0.15	3.19 ± 1.07
*Ruditapes decussatus*	2.68 ± 0.22	2.94 ± 0.36	2.83 ± 0.45	2.84 ± 0.1	2.84 ± 0.31

* A quantity of live bivalve molluscs collected from the same production area.
